# T101. ENRICHING PSYCHOTIC DISORDER CLASSIFICATION USING NATURAL LANGUAGE PROCESSING

**DOI:** 10.1093/schbul/sby016.377

**Published:** 2018-04-01

**Authors:** Rashmi Patel, Richard Jackson, Robert Stewart, Philip McGuire

**Affiliations:** King’s College London

## Abstract

**Background:**

Advances in molecular biology, genetics and neuroimaging have the potential to improve our understanding of psychotic disorders. However, the clinical classification of psychotic disorders has remained largely unchanged and is based on criterion-based diagnostic systems (such as ICD-10 and DSM-5) which do not necessarily reflect their underlying aetiology and pathophysiology. A more refined characterisation of clinical phenotype could help to improve our understanding of these disorders.

Clinical data are increasingly recorded in the form of electronic health records (EHRs). Automated information extraction methods such as natural language processing (NLP) offer the opportunity to quickly extract and analyse large volumes of clinical data from EHRs. We sought to characterise the range of presenting symptoms in a large sample of patients with psychotic disorders using NLP.

**Methods:**

Dataset: South London and Maudsley NHS Trust (SLaM) Biomedical Research Centre (BRC) Case Register comprising pseudonymised EHRs of over 270,000 people.

Clinical sample: 18,761 patients with an ICD-10 diagnosis of a psychotic disorders (F20, F25 or F31) and a control group of 57,999 patients with a non-psychotic disorder diagnosis (mood/affective/personality disorders without psychotic symptoms).

Data collection: The NLP software package TextHunter was used. All sentences containing keywords relevant to the following symptom categories were analysed using a support vector machine learning (SVM) approach: positive symptoms, negative symptoms, disorganisation, mania and catatonia. Data on 46 symptoms were obtained with 37,211 instances annotated to contribute training and gold standard data for machine learning. 2,950 instances were independently annotated to determine inter-annotator agreement.

**Outcomes:**

prevalence of psychotic symptoms and their association with ICD-10 diagnosis.

**Results:**

A good degree of inter-annotator agreement was achieved (Cohen’s κ: 0.83). Machine learning NLP achieved a mean precision (positive predictive value) of 83% and recall (sensitivity) of 78%. Among patients with psychotic disorders, the most frequently documented symptoms were paranoia, disturbed sleep and hallucinations. Psychotic symptoms were not limited to patients with an ICD-10 diagnosis of a psychotic disorder and were also present in the control group.

**Discussion:**

We found that psychotic symptoms were not limited to patients with a specific ICD-10 diagnosis and were present in a wide range of ICD-10 disorders. These findings highlight the utility of detailed NLP-derived symptom data to better characterise psychotic disorders.

